# Supplementary material for: Sediment discharge from Greenland’s marine-terminating glaciers is linked with surface melt
Source: Nat Commun. 2024 Feb 13;15:1332. doi: 10.1038/s41467-024-45694-1 (PMC10864362; doi:10.1038/s41467-024-45694-1)
Supplement: Supplementary file 1 — Supplementary Information [file 41467_2024_45694_MOESM1_ESM.pdf]

Supplementary information

Camilla S. Andresen, Nanna B. Karlsson, Fiammetta Straneo, Sabine Schmidt, Thorbjørn J. Andersen, Emily F. Eidam, Anders A., Nicolas Dartiguemalle, Laurence M. Dyke, Flor Vermassen and Ida G. Elkjær **Sediment discharge from Greenland's marine-terminating glaciers is linked with surface melt.** *Nature Communications.*

Source data file *Marine sediment data Greenland marine-terminating glaciers* is accompanying the paper and provided separately (Ref. 1)**:** Extended source data file with basic with basic Information for all cores, including core positions, mass accumulation rates, and glaciological data for glaciers contributing meltwater to the fjords from where the cores were taken (Data in Fig. 1, Fig. 2 and Table1). The file also includes a spreadsheet with the profiles of ^{210}Pb excess and ^{137}Cs activities for the marine sediment cores.

Mass accumulation rates based on ^{210}Pb and ^{137}Cs activities

The study presents newly calculated mass accumulation rates (MAR) from 27 marine sediment cores from fjords by Greenlandic marine terminating calving glaciers. All cores were obtained with coring devices (mainly Rumohr lot corer) which allow preservation of the core top. Locations of cores are shown in Supplementary Figure 4 and geographical coordinates provided in the source data file (ref. 1).

For the period 1950-1999 the MAR was estimated using water content and sedimentation accumulation rates (SAR):

Average MAR (kg m^{-2} yr^{-1}) = average SAR (m yr^{-1}) * dry bulk density (kg m^{-3}). Dry bulk density is calculated a ((100-average % water)/100) * 2650 kg m^{-3}, where 2650 kg m^{-3} is the density of quartz. The sediment water content was measured concurrently with sampling for ^{210}Pb analysis.

The naturally-occurring ^{210}Pb ($T_{1/2}$ = 22.3 years) is used to estimate sediment accumulation rates in the Greenland glacial fjords (Ref. 2 and 3). SAR is determined from profiles of ^{210}Pb in excess ($^{210}Pb_{xs}$) of that supported within sediment, by the decay of its radioactive parent (^{226}Ra), using the constant flux constant sediment (CF:CS) model. The compilation includes calculations of MAR on 19 cores for which profiles of $^{210}Pb_{xs}$ were previously reported (Source data file, ref. 1), and on 8 cores whose profiles of $^{210}Pb_{xs}$ are presented here (Fig. S1). To calculate SARs since the 1950s on the 27 cores, an iterative approach was used. A first SAR was calculated on the whole profile and an age model was established based on the sampling year of the cores. This dating then allowed to select the levels corresponding to sediment deposited since 1950 to recalculate average SAR for the period 1950-2009. A few of the cores only reach back to around 1980 (Source data file, Ref. 1) and for those the average accumulation rate for the entire core have been calculated.

Three labs (university of Copenhagen and Bordeaux, and Washington University) were involved in the determination of $^{210}Pb_{xs}$ on the 8 cores measured for this study: ER11-22, ER11-23, ER11-26, Cr01, Cr05, ER14, ER15 and ER16, all from Sermilik Fjord. Laboratory methodology used for determination of $^{210}Pb_{xs}$ on the remaining 19 cores is provided in the publications listed in the source data file (Ref. 1).

At the university of Copenhagen and Bordeaux, measurements on dry sediments were done using gamma spectrometers, that have permitted for determination of ^{210}Pb, ^{226}Ra and ^{137}Cs (for further details see Ref (4) and Ref (5). At Washington University, ^{210}Pb activities were determined by alpha spectrometry after a radiochemistry step according to the procedure described by Ref (2) and Ref (3). A mean supported ^{210}Pb activity was calculated as the average of ^{210}Pb determinations in deep sediment layers below the zone of ^{210}Pb exponential decline (between 67 and 203.5 cm for CR01 and between 48 and 188 cm for CR05). Note that age modeling was not performed on these cores due to concerns about calculating inventories in light of core 10 cm shortening of Cr01 after core retrieval.

Radionuclide activities are provided in the source data file (Ref. 1).

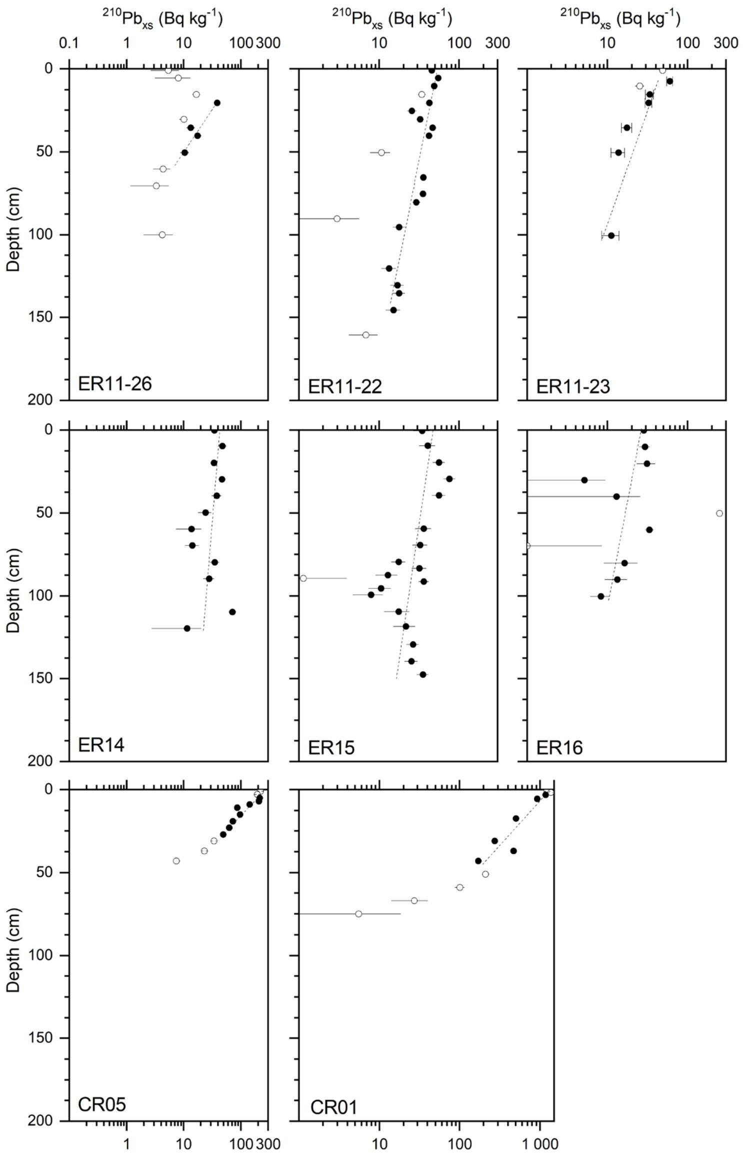

Supplementary Figure 1: ^{210}Pb excess profiles for eight Sermilik Fjord cores measured for this compilation, that were not previously published. The dark points correspond to the $^{210}Pb_{xs}$ values used to determine the sedimentation rates since 1950; the dotted line shows the corresponding regression.

Sedimentary Facies

The sediment cores in the analysis were assessed in terms of the sedimentary facies using x-rays and grain size analysis to omit turbidites (see source data file (ref. 1) for references to publications presenting the sedimentary facies of the cores providing input to the compilation). The cores, ER11-22, ER11-23, ER11-24, ER11-25, ER11-26, Cr01, Cr05, ER14, ER15 and ER16, ER07, ER11, ER13, all from Sermilik Fjord, are presented in Supplementary Figure 2 and 3.

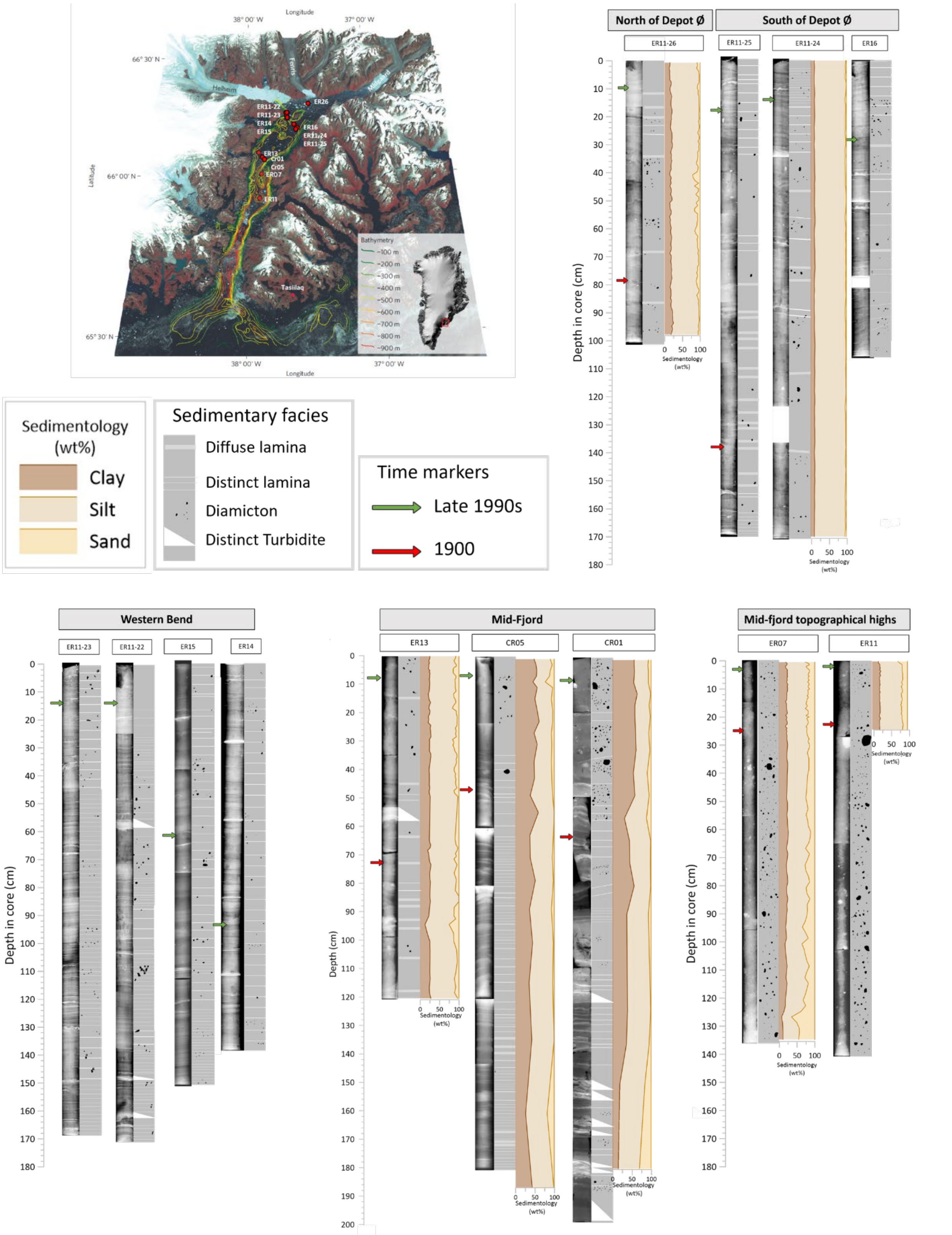

Supplementary Figure 2: X-ray, lithological logs and grain size distribution for Sermilik Fjord sediment cores. Core locations are shown on inset map. The bathymetry in inset map is a compilation of data presented in Ref (6). The background image is an oblique Landsat scene (L5231014_01419860911) draped over a digital elevation model.

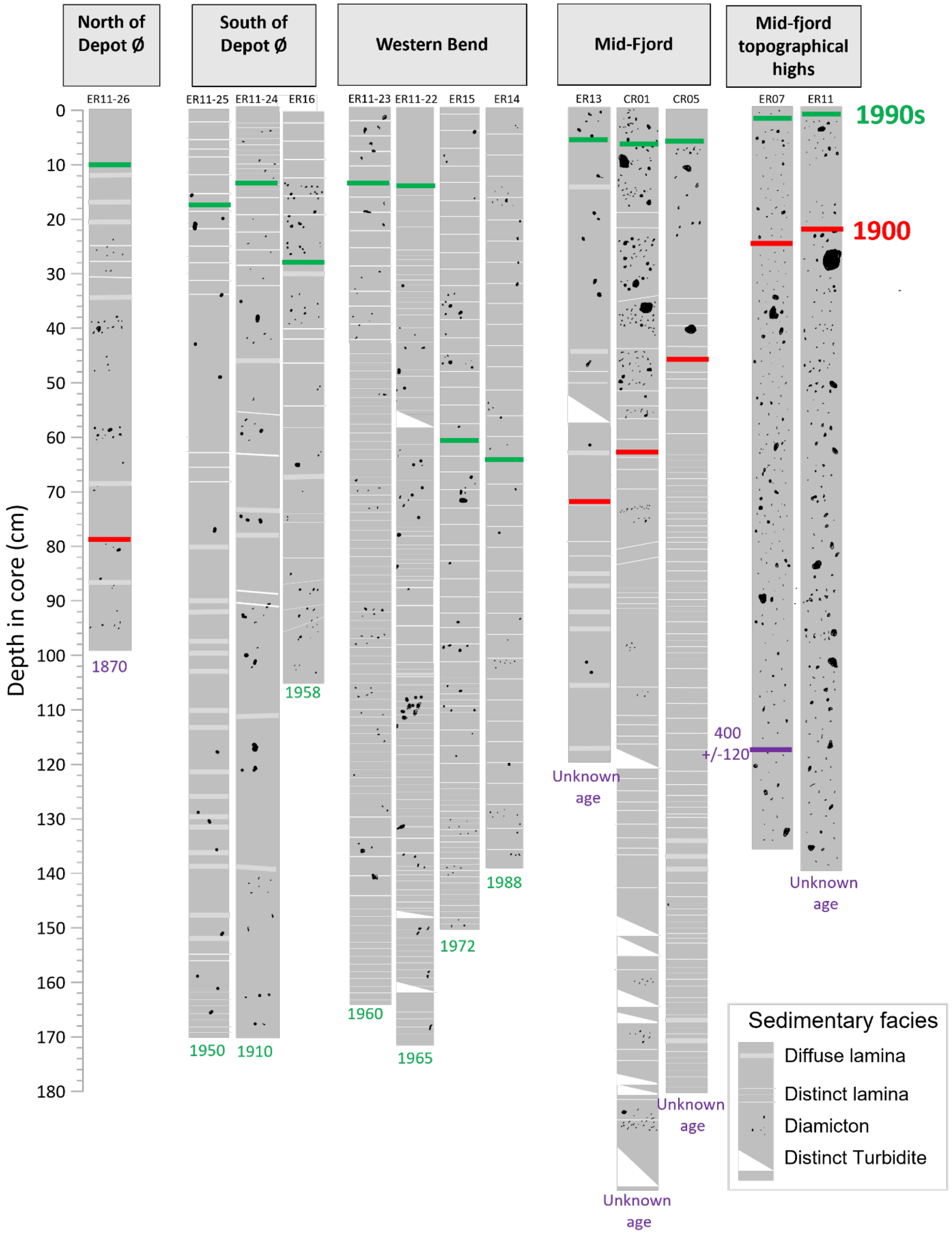

Supplementary Figure 3: Overview of all lithological logs from cores in Sermilik Fjords highlighting approximate isochrones (see Supplementary Figure 2).

Core locations

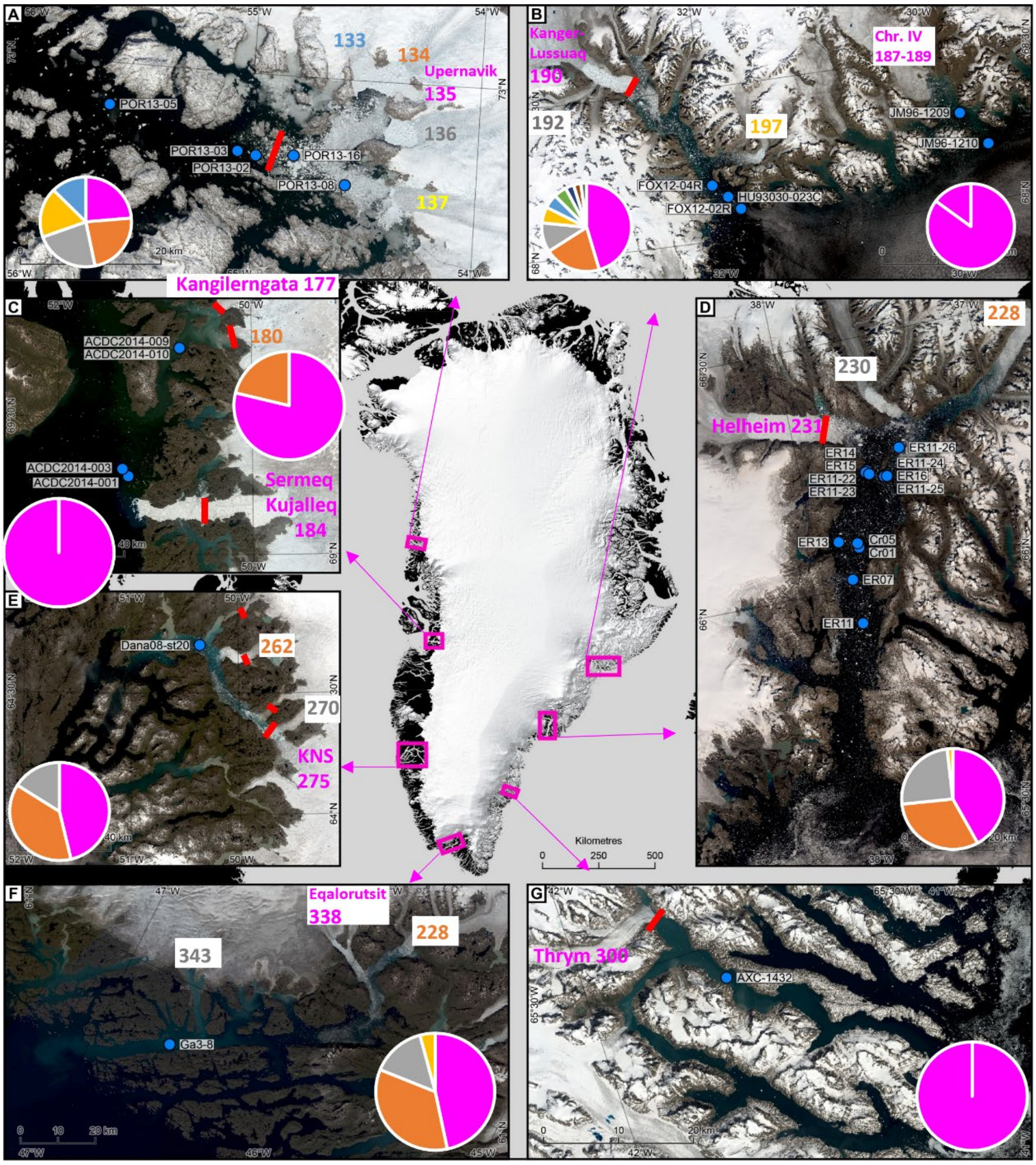

Supplementary Figure 4: Map of Greenland with inset maps for individual glacier-fjord systems from which sediment cores were obtained. The circle diagrams denote the relative surface melt partitioning between the individual glaciers terminating in the fjord. The numbers refers to the gates in Ref (7). **A**. Upernavik Glacier-fjord. **B**. Kangerlussuaq Glacier-fjord and Christian IV-Nansen Fjord. **C**. Kangilerngata Glacier-Ikerasak (Ata Sund) and Sermeq Kujalleq-Qeqertasuup Tunua (Disko Bugt). **D**. Helheim Glacier-Sermilik Fjord. **E**. KNS Glacier-Kangersuneq Fjord. **F**. Eqalorutsit Glacier – Ikersuaq Fjord. **G**. Thrym Glacier- Qimutuluittiip Kangertiva (Skjoldungen Fjord). Position of Little Ice Age margin is indicated with red line and references for publications presenting the exact margin positions are provided in the source data file accompanying the paper (Ref. 1). The central overview figure is composed of GIMP elevation data (Ref 8). Individual fjords are depicted with mosaics of pan-sharpened cloud-free Landsat 8 imagery collected in 2022.

Assessment of error based on core distance to glacier margin

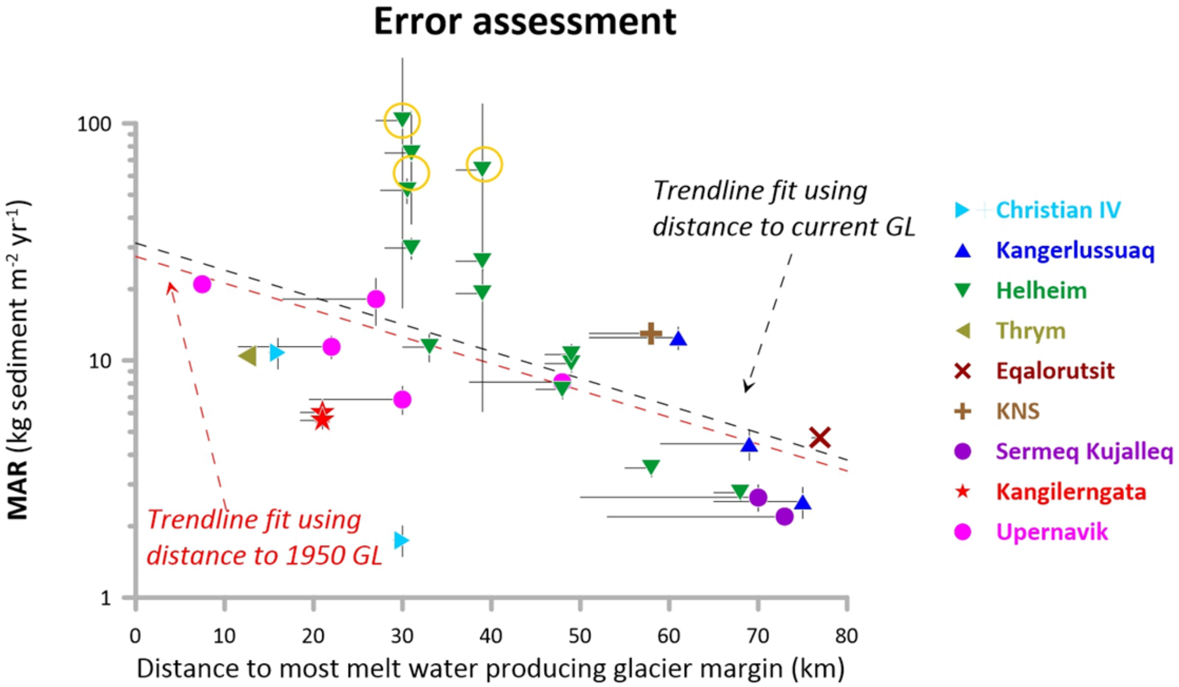

Supplementary Figure 5: Vertical and horizontal error assessments. Horizontal error is the distance between the current GL and the 1940 GL pointing in the direction towards the glacier margin. The vertical error is based on the maximum error in the sedimentation rate. The three cores with high error (ER14, ER15 and ER16) are marked with yellow circle.

Supplementary References

1. Andresen, Camilla S; Karlsson, Nanna B.; Straneo, Fiammetta; Schmidt, Sabine; Andersen, Thorbjørn Joest; Eidam, Emily F.; et al. (2024). Table S1 Marine sediment data by Greenland marine-terminating glaciers. figshare. Dataset. https://url12.mailanyone.net/scanner?m=1rSFyv-0000tn-5Q&d=4%7Cmail%2F90%2F1706013000%2F1rSFyv-0000tn-5Q%7Cin12f%7C57e1b682%7C15209072%7C14343128%7C65AFB24D3F2E394FC3430FBDFF6096E6&o=%2Fphto%3A%2Fdts0ri.6%2F1.ogf%2F08g9.i4m3rsh5.22ae1634&s=CBsQiNJHaeLukXrxNeTv3TxjUfE
2. Jaeger, J.M., and C.A. Nittrouer, 1999. Marine record of surge-induced outburst floods from the Bering Glacier, Alaska. Geology, 27 (9), 847-850.
3. Jaeger, J.M., and C.A. Nittrouer, 1999. Sediment deposition in an Alaskan fjord: Controls on the formation and preservation of sedimentary structures in Icy Bay. Journal of Sedimentary Research, 69, 1011-1026.
4. Andersen, T.J., 2017. Some practical considerations regarding the application of 210 Pb and 137 Cs dating to estuarine sediments. Applications of paleoenvironmental techniques in estuarine studies, pp.121-140.
5. Schmidt S. & De Deckker P. 2015. Present-day sedimentation rates on the southern and southeastern Australian continental margins. Australian Journal of Earth Sciences 62, 143-150, doi: 10.1080/08120099.2015.1014846

6. Andresen, C. S., Straneo, F., Ribergaard, M. H., Bjørk, A. A., Andersen, T.J., Kuijpers, A., Nørgaard-Pedersen, N., Kjær, K. H., Schjøth, F., Weckström, K. and Ahlstrøm, A. P. 2012. Rapid response of Helheim Glacier in Greenland to climate variability over the past century. Nature Geoscience 5, 37-41, doi:10.1038/ngeo1349.
7. Mankoff, K. D., Noël, B., Fettweis, X., Ahlstrøm, A. P., Colgan, W., Kondo, K., ... & Fausto, R. S.. Greenland liquid water discharge from 1958 through 2019. *Earth System Science Data*, *12*(4), 2811-2841. (2020). https://doi.org/10.5194/essd-12-2811-2020
8. Howat, I. M., Negrete, A., and Smith, B. E. (2014) The Greenland Ice Mapping Project (GIMP) land classification and surface elevation data sets, The Cryosphere, 8, 1509–1518.
